# Changes in Activity Participation Among Older Adults With Subjective Cognitive Decline or Objective Cognitive Deficits

**DOI:** 10.3389/fneur.2019.01393

**Published:** 2020-01-15

**Authors:** Shlomit Rotenberg, Adina Maeir, Deirdre R. Dawson

**Affiliations:** ^1^Dawson Lab, Rotman Research Institute, Baycrest, Toronto, ON, Canada; ^2^Cog-Fun Lab, School of Occupational Therapy, Hebrew University, Jerusalem, Israel; ^3^Department of Occupational Science & Occupational Therapy, University of Toronto, Toronto, ON, Canada

**Keywords:** metamemory, daily functioning, activity participation, aging, subjective cognitive decline

## Abstract

Participation in daily activities is crucial for healthy aging. There is limited research on participation of older adults with subjective cognitive decline (SCD), defined as the experience of cognitive deficits with no evidence of objective cognitive deficits. Therefore, this study examined perceived changes in participation in this population, and compared it to perceived changes reported by individuals with objective cognitive deficits. The study aimed to: (1) examine the reported changes in activity participation of older with SCD; (2) investigate differences in the reported changes in participation between individuals with SCD and those with mild or severe objective cognitive deficits; (3) examine the relationship between activity participation, subjective memory, and objective cognitive status; and (4) explore whether subjective memory explains additional variance in activity participation after accounting for age and objective cognitive deficits. Participants were 115 older adults (60+), divided into three groups based on their Montreal Cognitive Assessment (MoCA) scores: (1) SCD (MoCA≥26; *n* = 66); (2) mild objective cognitive deficits (MoCA = 20–25; *n* = 34); and (3) severe objective cognitive deficits (MoCA ≤ 19; *n* = 15). The Activity Card Sort was used to measure participation in instrumental activities of daily living, social, and leisure activities. The Multifactorial Memory Questionnaire—Ability subscale was used to assess subjective memory. We found that individuals with SCD, mild cognitive deficits and severe cognitive deficits reported participation withdrawal to a level of 80, 70, and 58% of their past participation, respectively. A significant between group difference was found on participation [χ^2^(2) = 16.44, *p* < 0.01], with the SCD group reporting higher participation than the other two groups. Participation significantly correlated with both cognitive status (*r* = 0.40, *p* < 0.01) and subjective memory (*r* = 0.45, *p* < 0.05). A regression analysis revealed that subjective memory contributed significantly to the explained variance in participation, beyond that accounted for by objective cognitive deficits and age. Our findings demonstrate the important role of subjective memory problems in activity participation of older adults, even in the absence of objective cognitive deficits.

## Introduction

Many older adults experience cognitive problems. The reported prevalence of subjective cognitive problems in older adults varies widely, from 22 to 80% (1–3). The prevalence of objective impairments is significantly lower, with an estimated 6–26% of older adults diagnosed with mild cognitive impairment (4, 5), and 4–15% with dementia (5, 6). The subjective experience of cognitive decline without objective evidence of cognitive deficits is referred to as subjective cognitive decline (SCD) (7). SCD is increasingly understood to be a risk factor for future cognitive decline (8, 9) and considered by some to be prodromal for dementia (10).

The relationship between objective and subjective cognitive functioning in older adults is not fully understood. A systematic review and meta-analysis of 50 studies showed a small but significant relationship between subjective and objective cognitive functioning in older adults (11). Additionally, subjective memory, measures by the reported frequency of memory related mistakes in daily functioning, was associated with objective cognitive abilities in older adults with mild cognitive impairment (MCI), but not in older adults with SCD (12).

Participation in daily activities of older adults with SCD is not often studied. This is important because preserving functional abilities despite age related health changes is crucial for healthy aging as defined by the World Health Organization (WHO) (13). Moreover, the International Classification of Functioning, Disability and Health (ICF) (14), that describes the broad consequences of health conditions, highlights the important role of participation in daily activities for the well-being of individuals with any health condition. The ICF model describes reciprocal interactions between body functions, such as objective cognitive abilities, contextual personal factors such as subjective perceptions of memory and participation in everyday life activities (14). Despite the importance of activity participation in aging and the theoretical basis, provided by the ICF, for interactions between participation, cognition status and subjective memory, this relationship has not been widely studied among older adults with SCD.

The few studies that explored whether older adults with SCD report difficulties with everyday functioning have focused predominantly on basic and instrumental activities of daily living (BADL and IADL). A population based study in Germany found that only 3.4% of older adults with SCD reported impaired IADL (15), suggesting that people with preserved cognitive abilities are fairly independent in IADL. Furthermore, older adults with SCD reported better IADL functioning compared to older adults with MCI and dementia (16). However, older adults with SCD are more likely to develop BADL and IADL difficulties over a 1 year period compared to healthy older adults with no SCD (17). Additionally, conversion rates from SCD to dementia are higher in people with SCD who also have impaired IADL (15).

The ICF definition of participation as “involvement in a life situation” [(14), p. 10], covers a wide range of domains, including IADL, community, social, recreational, leisure, and religious activities. In line with the ICF model, this study aimed to expand on the body of knowledge regarding activity participation in older adults, and examined their participation not only in IADL activities, but also in a wide range of social and leisure activities. This is important because participation in social and leisure activities is associated with decreased risk for future cognitive decline in older adults (18, 19). Due to the limited research on activity participation of older adults with SCD, this study aimed to understand participation in this population by examining perceived changes in participation over 5–10 years, and comparing it to changes reported by people with objective cognitive deficits. We also aimed to understand the relationship between subjective memory, objective cognitive status, and activity participation. The specific study objectives were to: (1) examine perceived changes in participation of older adults with SCD and those with objective cognitive deficits in relation to their own participation 5–10 years before; (2) investigate differences in participation withdrawal between older adults with SCD and those with objective cognitive deficits; (3) examine the correlations between participation, subjective memory, and objective cognitive status; and (4) explore whether the severity of subjective memory problems explains variance in participation, over and above that explained by age and objective cognitive deficits.

## Materials and Methods

### Study Design and Procedure

This study was a secondary data analysis of data collected for two studies: (1) a pilot intervention study for older adults with SCD (pre-training data only), performed in Canada (20); and (2) a cross-sectional study comparing older adults who reported memory problems at a geriatric clinic to age matched older adults who did not seek medical help for their perceived memory problems, performed in Israel (21). In study 1, participants were recruited from a research subject pool and a community psycho-education program, and in study 2 through a geriatric clinic and convenience sampling in the community. The use of the data for this secondary analysis was approved by the Baycrest Research Ethics Board (study 1), the Helsinki Committee of Maccabi Healthcare Services and the Hebrew University Institutional Review Board (study 2).

### Participants

Participants were 115 community dwelling older adults, age 60, or greater. Participants from both studies had self-reported memory and/or cognitive problems. Participants were allocated to one of three groups based on their cognitive status, as measured by the Montreal Cognitive Assessment (MoCA) (22), using cut-off scores suggested by Horton et al. (23): (1) SCD, with MoCA scores ≥ 26 (*n* = 66); (2): mild objective cognitive deficits (mild-CD), with MoCA scores = 20–25 (*n* = 34); and (3): severe cognitive deficits (severe-CD), with MoCA scores ≤ 19 (*n* = 15). According to Horton et al. (23) the mild-CD and severe-CD groups may be perceived as equivalent to MCI and dementia, respectively, however, the available data were not sufficient to inform such diagnoses.

### Measures

Cognitive status was measured using the MoCA (22), a widely used short cognitive screening test that covers a wide range of cognitive domains. The MoCA scores range from 0 to 30, with higher scores reflecting better cognitive performance. The MoCA demonstrated good internal consistency (Cronbach's α = 0.83) and adequate known-group validity, with high diagnostic value in older adults with MCI and dementia (24).

The Activity Card Sort (ACS) (25) is a self-report measure that examines current activity participation compared to an individual's past participation. The ACS examines participation in four domains: (1) IADL (e.g., laundry, paying bills); (2) leisure with low physical demands (e.g., hand crafts, watching television, attending concerts); (3) leisure with high physical demands (e.g., sports, camping); and (4) social activities (e.g., entertaining, volunteer work). The ACS is comprised of pictures representing a wide range of activities, each scored on involvement in the past (rated as “performed” or “didn't performed”) and in the present (rated as “doing now,” “doing less than in the past,” or “not doing/have given up”). Each activity performed in the past is given a score of one point, and a total score of past participation is calculated as the sum of those activities. Current activities are allocated one point if currently performed, 0.5 points if performed less than in the past, and zero points for activities that are not currently performed. These scores are summed to produce a current participation score. Changes in participation are than calculated as the proportion of the activities currently performed relative to those performed in the past, by dividing the total current participation score by the total past participation score. The ACS provides a total change score, and four sub-scores for four activity domains. Scores range from 0 to 100%, with lower percentages scores representing less preserved activity participation and more withdrawal from previous participation. The original ACS version, with 80 pictures, was used in study 1, and the Hebrew version (26), with 88 pictures, was used in study 2. Participants were asked to consider “past participation” as their participation five (study 1) or 10 (study 2) years earlier. The ACS presents good known group validity, and was able to discriminate between healthy adults, healthy older adults, and individuals with neurological disabilities (26, 27).

The Multifactorial Memory Questionnaire (MMQ)—Ability subscale (28) was used to assess subjective memory. The MMQ-Ability is a self report questionnaire that measures the frequency of memory related mistakes in daily life (e.g., forget to pay a bill, difficulty recalling a word). It consists of 20 items, scored on a five point Likert scale. The total score ranges from 0 to 80, with higher scores indicating better subjective memory ability. The MMQ-Ability has good internal consistency (Cronbach's α = 0.93) and excellent content validity (83–100% agreement between raters) in clinical and non-clinical older adult populations (29). Construct validity of the MMQ-Ability was moderate to strong (*r* = 0.43–0.89) with other subjective memory questionnaires (29). Data on the MMQ were available for participants in study 2 only (*n* = 91).

Each of the two studies in this secondary data analysis used a different measure of mood. Study 1 used the Center for Epidemiological studies Depression scale (CES-D) (30), a 20 item questionaire of depression symptomology, scored on a four point scale. Study 2 (*n* = 91) used the Patient Health Questionnaire (PHQ-9) (31), a nine item questionnaire scored on a four point scale. For both measures, higher scores reflect more depressive symptomology. We classified participants as having depressive symptomology based on the accepted cutoff score of 16 for the CES-D (30) and 10 for the PHQ-9 (31).

### Statistical Analysis

Descriptive statistics were used to describe the demographic variables and changes in participation as rated on the ACS. The Kruskal-Wallis H test was used to compare between the three cognitive groups on demographic data and the level of maintained participation as measured by the ACS. A non-parametric test of between group differences was chosen because four of the five ACS scores were not distributed normally. Where statistically significant differences were found in the Kruskal-Wallis H test, we performed a Dunn's *post-hoc* test with Bonferroni correction, to compare each of the three cognitive groups to the others. A chi-square test was used to compare between the cognitive groups on nominal variables (gender and presence of depressive symptoms). Effect sizes were calculated using epsilon square (ε^2^) (32), and interrupted as follows: ε^2^ = 0.00<0.01-negligible; 0.01<0.04-weak; 0.04<0.16-moderate; 0.16<0.36-relatively strong; 0.36<0.64-strong; 0.64<1.00-very strong (33). A partial correlation was computed between participation, objective cognitive status and subjective memory, controlling for age. An exploratory hierarchical linear regression model was used to assess the impact of subjective memory on participation, beyond age and objective cognitive functioning. Age and MoCA scores were entered into blocks one and two, and the MMQ-Ability score was added into the third block. The reported *p* values are the result of two-sided tests, with an alpha level of 5%. All analyses were performed using SPSS 24.0 for Windows (SPSS Inc., Chicago, IL).

## Results

The demographic characteristics of the full sample and by cognitive groups are presented in Table 1. Significant group effects were found for age, education and depression. *Post-hoc* tests revealed that the SCD group was significantly younger and more educated than the two groups with objective cognitive deficits, but no significant differences were found between the mild-CD and severe-CD groups on age and education. The severe-CD group significantly more depressed than the other two groups.

**Table 1:** Participant characteristics and between group comparison.

	**Total Sample (*n* = 115)**	**SCD (*n* = 66)**	**Mild-CD (*n* = 34)**	**Severe-CD (*n* = 15)**	
	***n* (%)**	***n*** **(%)**			****χ^2^(2)**^a^, *p***
Gender-Female	74 (65.2)	47 (71.2)	20 (58.8)	8 (53.3)	2.59 (0.274)
Depressive symptoms^b^	14 (12.2)	4 (6.1)	3 (8.8)	8 (53.3)	19.36 (0.000)
	**Mean** **±** **SD**	**Mean** **±** **SD**			**χ^2^(2)^c^**
Age (years)	77.88 ± 7.15	75.86 ± 7.49	80.00 ± 7.56	81.93 ± 6.50	12.41 (0.002)
Education (years)	15.20 ± 3.99	15.96 ± 3.65	14.28 ± 4.36	14.00 ± 4.16	7.66 (0.020)
MMQ-Ability^d^	43.18 ± 13.20	46.80 ± 12.89	41.53 ± 12.24	36.07 ± 13.30	9.65 (0.008)
**ACS**					
Total Score	73.37 ± 16.79	78.57 ± 13.74	70.05 ± 15.99	57.99 ± 20.22	16.44 (0.000)
IADL	79.40 ± 19.03	84.28 ± 13.99	77.16 ± 21.42	63.07 ± 23.39	10.94 (0.004)
Leisure, low physical demands	80.20 ± 15.97	82.83 ± 13.50	79.41 ± 17.37	70.43 ± 19.53	5.76 (0.056)
Leisure, high physical demands	53.15 ± 29.37	61.68 ± 26.42	46.43 ± 27.21	30.86 ± 32.47	14.16 (0.001)
Social	71.65 ± 20.08	77.11 ± 17.91	67.14 ± 17.87	57.86 ± 25.41	12.39 (0.002)

*SCD, subjective cognitive decline; CD, cognitive deficits; MMQ, Multifactorial Memory Questionnaire; ACS, Activity Card Sort*.

a *Chi-square test*.

b *Presence of depressive symptoms was determined by the cut-off scores of 16 for the Center for Epidemiological studies Depression scale (CES-D) for participants in study 1, and 10 on the Patient Health Questionnaire (PHQ-9) for participants in study 2*.

c *Kruskal-Wallis Test*.

d *MMQ scores available for 91 participants only*.

To examine perceived changes in participation (study objective 1) we calculated the proportion of self-reported current participation in relation to individuals past participation. Individuals with SCD, mild-CD and severe-CD all reported reduced levels of participation of 79, 70, and 58%, respectively (see Table 1). We found similar trends of reduced participation in all four ACS subscales, with the SCD group reporting highest rates, and the severe-CD group reporting lowest rates of retained participation.

**Figure 1:**
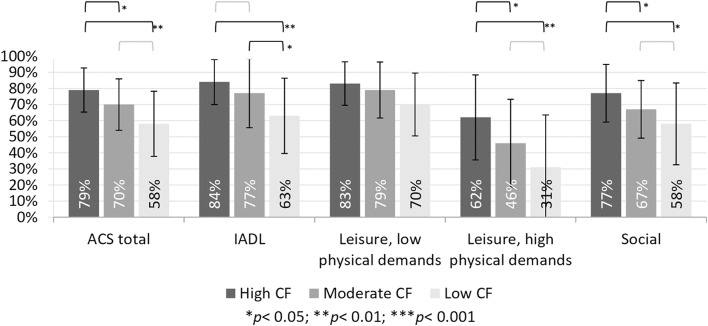
Between group differences on participation: *post-hoc* Dunn's test on ACS scales.

To address objective 2, we examined between group differences in the reported changes in participation using a Kruskal-Wallis test. The results, presented in Table 1, show a significant group effect on participation reported on the ACS-total scores and three of the four ACS sub-scores. A *post-hoc* Dunn's test showed that the SCD group reported significantly higher retained participation compared to both other groups on the ACS-total, as well as the subscales of social activities and leisure activities with high physical demands (see Figure 1). The SCD group reported significantly higher retained participation in IADL compared to the severe-CD group, but not to the mild-CD group. The mild-CD group reported significantly higher retained participation compared to the severe-CD group on the IADL subscale. The between group effect size on the ACS-total score was moderate (ε^2^ = 0.07) between the SCD and mild-CD groups, and relatively strong (ε^2^ = 0.17) between the SCD and severe-CD groups.

For objective 3, associations of the ACS scores to objective cognitive status (measured by the MoCA) and subjective memory (MMQ-Ability) were examined using a partial correlation analysis. Age was defined as a covariant because the SCD group was significantly younger than the other two groups. Following application of the Bonferroni correction, we found significant positive moderate correlations between the ACS total score and both MoCA scores and MMQ-Ability scores, when controlling for age (see Table 2). The results suggest that more preserved participation is associated with better objective cognitive status and lower frequency of memory problems in daily life (reflected by a higher MMQ-Ability score).

**Table 2:** Associations of participation to objective cognition and subjective memory.

	**MoCA**	**MMQ—Ability^a^**
	***n* = 115, r^a^ (*p*)**	***n* = 91, r (*p*)^b^**
ACS—Total score	0.40 (0.000)	0.45 (0.000)
ACS—IADL	0.39 (0.000)	0.34 (0.001)
ACS—Leisure, low physical demands	0.27 (0.010)	0.35 (0.001)
ACS—Leisure, high physical demands	0.29 (0.006)	0.42 (0.000)
ACS—Social	0.27 (0.012)	0.36 (0.000)

*MoCA, Montreal Cognitive Assessment; MMQ, Multifactorial Memory Questionnaire; ACS, Activity Card Sort; IADL, instrumental activities of daily living*.

a *MMQ scores available for 91 participants only*.

b *Correlations were calculated with age as a covariate to control for age differences*.

We used an exploratory hierarchical linear regression model to examine whether the severity of subjective memory problems explains additional variance in participation over and above that explained by age and objective cognitive (objective 4). Age was entered in block one, the MoCA was added in block two and the MMQ-Ability was added into the third block. The regression results (see Table 3) show that age and MoCA scores explained a significant proportion of the variance (33.2%) of participation. After controlling for age and cognitive status, subjective memory significantly explained another 9% of the variance in participation. The overall model explained 42.3% of the variance in participation among older adults.

**Table 3:** Exploratory hierarchical regression for participation (*n* = 91).

	**B**	**SE B**	**Beta (*p*)**	***R*^**2**^**	***R*^**2**^ change**	**F (*p*)**
**Block 1**						
Age	−1.11	0.23	−0.45 (0.000)	0.21	0.21	23.11 (0.000)
**Block 2**						
Age	−0.86	0.22	−0.35 (0.000)	0.33	0.13	16.65 (0.000)
MoCA	1.84	0.45	0.37 (0.000)			
**Block 3**						
Age	−0.94	0.21	−0.38 (0.000)	0.42	0.09	13.61 (0.000)
MoCA	1.33	0.44	0.27 (0.004)			
MMQ-Ability	0.43	0.12	0.32 (0.000)			

*MoCA, Montreal Cognitive Assessment; MMQ, Multifactorial Memory Questionnaire; ACS, Activity Card Sort*.

## Discussion

This study examined perceived changes in participation in IADL, leisure, and social activities among older adults with SCD and those with objective cognitive deficits. We found that older adults with SCD reported reduced activity participation, to ~79% of their total participation 5–10 years prior. Although they reported significantly less withdrawal from participation compared to both the mild-CD (70%) and the severe-CD (58%) groups, the reported withdrawal from their own level of participation warrant attention. We also examined the relationship of perceived changes in participation to subjective memory and objective cognition, and found significant moderate to strong correlations. The severity of subjective memory problems explained an additional 9% of the variance in participation, beyond the 33% explained by objective cognitive deficits.

### Participation in Older Adults With SCD

Participants with SCD reported withdrawal from social activities (77%) and leisure activities with high physical demands (62%), and to a lesser extent also from IADL (84%) and leisure activities with low physical demands (83%). While there are no normative data available for the ACS, these findings are concerning as we know that engagement in social and leisure activities is important for delaying and preventing cognitive decline as people age (18). The reported decline in leisure activities with high physical demands are specifically disturbing given the association between aerobic activity and preserved cognitive functioning in older adults (34).

Although the reported withdrawal from participation in those with SCD was less severe than in the two groups with objective cognitive deficits, the results suggest that healthcare professionals should assess participation in IADL, social, and leisure activities among older adults reporting memory problems, even in the absence of objective cognitive deficits. Identifying withdrawal from participation in people with SCD is important, since activity participation is a modifiable factor, that was shown to improve through intervention (35, 36). Preventing participation withdrawal in older adults with subjective memory problems is key to supporting their quality of life (21). Also, given the higher rates of conversion from SCD to dementia in people who also report impaired IADL (15) it is possible that identifying these impairments and providing interventions that improve IADL functioning in people with SCD may delay their future cognitive deterioration.

The reported withdrawal from social and leisure activities in the SCD group to a level of 62–82% of previous participation is important to highlight because most studies on this population focus on ADL and/or IADL and not much is known about changes their social and leisure participation. The reported reduction in social and leisure activities, especially leisure activities with high physical demands, is disturbing because involvement in social activities and other activities in the community were shown to be associated with lower risk of cognitive decline over a 3–4 year period (18, 19).

### Participation and Objective Cognition

The moderate and significant correlations between participation and objective cognitive status in our sample of older adults who report subjective memory problems (with or without objective cognitive deficits), suggests that objective cognition plays a role in their everyday functioning. This is supported by the finding that those with no objective cognitive deficits reported significantly less occupational withdrawal than both other groups.

Examination of the four ACS subscales revealed a consistent pattern over the three cognitive groups, where the most withdrawal is reported from leisure activities with high physical demands, followed by social activities. The SCD group reported significantly less withdrawal from participation in both these areas compared to the other two groups. The mild-CD and severe-CD groups did not differ significantly on participation in leisure activities with high physical demands and social activities. This could be explained by the high cognitive demands inherent in social activities and leisure activities with high physical demands. On the ACS, both sub-scales include non-routine activities that require planning and problem solving, and therefore may be more susceptible to decline in people with cognitive deficits. However, it is also possible that physical ability contributed to these scores as many of the activities in these two subscales are performed outside the home. As the SCD group is significantly younger than the other two groups, it is possible that they are more mobile. Leisure activities with low physical demands were reported to be relatively preserved in all three groups. It is possible that these activities are more preserved not only due to their low physical demands, but also do to their low cognitive demands, because many of the activity in this sub-scale of the ACS are non-complex leisure activities, such as watching movies and television or doing hand crafts. We would suggest that future studies document physical ability to help elucidate this issue, and provide insight as to the underlying physical and/or cognitive mechanisms behind the reported withdrawal from participation.

The severe-CD group reported significantly more withdrawal in IADL compared to the other two groups, yet there was no statistically significant difference between the SCD and mild-CD groups. These findings support the definition of “major neurocognitive disorder” (previously dementia), as involving interference in everyday functioning and independence (37). There are two ways to explain the relatively preserved IADL participation in the mild-CD group. One is that many of the IADL activities in the ACS are routine activities (e.g., doing dishes, laundry) that don't require high cognitive reserves. Another possible explanation is that IADL activities, more than social and leisure activities, are fundamental in preserving independence. Therefore, when everyday functioning requires greater effort due to reduced cognitive abilities (38), it is possible that these efforts are channeled toward IADL activities at the expense of social and leisure activities.

### Participation and Subjective Memory

An important finding from this study is that subjective memory explained 9% of the variance in participation in everyday activity, in addition to that explained by age and objective cognition. Identifying difficulties in everyday functioning in people with subjective memory problems, even in the absence of objective cognitive decline (i.e., people with SCD) is important, both as a risk factor for future cognitive decline (15) and as a factor that may influence their current quality of life (21, 39). Healthcare professionals should assess participation from a broad perspective, and inquire about changes in social and leisure participation, even in people with no objective cognitive deficits.

We found that subjective memory was moderately and significantly correlated with participation in daily activities, when controlling for age. The more memory related mistakes older adults reported in their everyday life, the more withdrawal they reported from participation daily activities. Similar findings have been reported in qualitative studies of older adults who experience cognitive problems including declines in daily activities, changes in life roles and loss of independence (40, 41). Similar to Montejo et al. (42), we found a significant relationship between subjective memory and IADL functioning. Our results expand this body of knowledge and show significant associations between subjective memory and social and leisure activities, areas of daily activities sparsely studied in this population.

## Conclusion

SCD is not known to be associated with decline in daily functioning (15), but this study suggests that although people with SCD are independent in BADL and IADL they experience withdrawal from social and leisure activities. The results highlight the importance of asking people with SCD about change in their participation, to indentify withdrawal from participation early on in the potential trajectory of cognitive decline, and provide intervention to promote ongoing participation.

## Study limitations

This study had a number of limitations. First, we were unable to control for depression in the regression analysis as there were too few people over the cut-off score for depression to do a sub-group analysis. This may have affected the results, as depression is known to interact with cognition in this population (11). While it has been argued that understanding the day-to-day difficulties experienced by people with subjective cognitive problems is clinically important regardless of the etiology (11), we think this warrants further study. We also did not have a measure of apathy, another factor that may have had a mediating effect on participation. A second limitation is that participants' cognitive status was determined based only on the MoCA test, which is not a comprehensive diagnostic tool (22). Although we used cut-off scores suggested by Horton et al. (23), we did not have the necessary recourses to make conclusive diagnoses of SCD, MCI or dementia in our study participants. Thus, the division of our groups may not be a completely accurate representation of these three diagnoses. Our use of the MMQ-Ability subscale as the measure of subjective cognition also may have provided a limited representation of broader concept of SCD. In future studies, we suggest using other measures of subjective cognition and adding a control sample of healthy older adults who report no subjective cognitive changes, in order to compare changes in activity participation related to normal aging with those that we observed in individuals with SCD and objective cognitive changes. A third limitation of the study is that the analysis was unable to account for the different recruitment methods in the two parent studies. The relationship between subjective and objective cognition may differ in older adults recruited through community sources compared to those recruited in a clinical context (43). Finally, several methodological issues make us cautious about generalizing the results. The use of convenience sampling in both studies means that the samples may not be representative of the larger population; the self-report nature of the MMQ and ACS means these data are subject to recall bias; and finally, we had MMQ scores on only 91 of the participants.

## Data Availability Statement

The raw data supporting the conclusions of this manuscript will be made available by the authors, without undue reservation, to any qualified researcher.

## Ethics Statement

The studies involving human participants were reviewed and approved by Baycrest Research Ethics Board; the Helsinki Committee of Maccabi Healthcare Services; and the Hebrew University Institutional Review Board. The patients/participants provided their written informed consent to participate in this study.

## Author Contributions

SR, AM, and DD contributed to the conception and design of the study. SR organized the database, performed the data analysis, and wrote the manuscript. AM and DD contributed to data analysis and interpretation. DD revised the manuscript. All authors contributed to manuscript revision, read and approved the submitted version.

### Conflict of Interest

The authors declare that the research was conducted in the absence of any commercial or financial relationships that could be construed as a potential conflict of interest. The reviewer MB and handling editor declared their shared affiliation at the time of the review.
